# PROTEASOME DYSFUNCTION A KEY PATHOLOGIC FEATURE OF ALZHEIMER’S DISEASE MITIGATED BY PROTEASOME ACTIVATORS

**DOI:** 10.1093/geroni/igae098.4250

**Published:** 2024-12-31

**Authors:** Andrew Pickering

**Affiliations:** University of Texas Health Science Center at Houston, Houston, Texas, United States

## Abstract

Alzheimer’s Disease (AD) is the leading cause of dementia worldwide, affecting nearly 50 million people and characterized by cognitive decline, β-amyloid plaques, and hyperphosphorylated tau. The failure of numerous clinical trials targeting β-amyloid (Aβ) underscores the need for novel therapeutic strategies. A consistent feature of AD, particularly in the hippocampus, is reduced proteasome activity. This impairment is thought to result from direct inhibition by Aβ or hyperphosphorylated tau, disrupting essential neuronal processes like memory and synaptic plasticity. This study explores the hypothesis that AD-related deficits are driven, in part, by Aβ-induced proteasome dysfunction. We demonstrate that oligomeric Aβ inhibits 20S proteasome activity while destabilizing the 26S proteasome into free 20S units. Treatment with proteasome activators enhances 20S and 26S function, reducing Aβ42-induced toxicity in SK-N-SH cells. In Drosophila overexpressing Aβ42, oral administration of proteasome agonists delayed mortality and restored cognitive function. Similarly, in mouse models, chronic treatment with proteasome activators protected against Aβ42-induced working memory deficits, and acute treatment significantly improved spatial learning deficits in hAPP(J20) mice. Our findings reveal that Aβ exerts dual inhibitory effects on 20S and 26S proteasomes, contributing to AD pathology. Proteasome activation mitigates these deficits, suggesting that enhancing proteasome activity could be a promising therapeutic approach for AD.

